# Next-generation sequencing yields the complete mitogenome of Niya chicken (*Gallus gallus*)

**DOI:** 10.1080/23802359.2020.1791754

**Published:** 2020-07-15

**Authors:** Jingjing Gu, Sheng Li

**Affiliations:** a College of Animal Science and Technology, Hunan Agricultural University, Changsha, China; b Hunan Key Laboratory for Genetic Improvement of Animals, Changsha, China; c Hunan Engineering Research Center of Poultry Production Safety, Changsha, China; d Maxun Biotechnology Institute, Changsha, China

**Keywords:** Niya chicken, complete mitogenome, next-generation sequencing

## Abstract

In this study, the complete mitochondrial genome sequence of Niya chicken (Gallus gallus) was obtained by using the next-generation sequencing technology for the first time. The complete mitogenome sequence of Niya chicken is 16,784 bp in length, containing 2 ribosomal RNAs, 13 protein-coding genes, 22 transfer RNA genes and a D-loop region. Our work provides a valuable source of data for phylogenetic studies and helps us to understand the maternal origins of the native chickens.

The existence of Niya chicken has not been reported until 2006 during a national livestock resources survey in China. Later in 2010, this newly discovered breed was listed on the National Livestock and Poultry Genetic Resources Protection List of China based on its unique characteristics. Niya chickens have uniformly black feathers and are believed to have been bred by the local people in the Taklimakan Desert region, giving them a long history of living in a hot, dry, harsh environment. To understand the maternal origin of the Niya chicken, we assembled its complete mitochondrial genome using high-throughput sequencing technology for the first time. The Niya chicken was collected from its core breeding center – Minfeng County (37.07 N and 82.70 E), Xinjiang Uygur Autonomous Region, China. The Niya chicken specimen (Voucher No. NY150159) was stored at −80 °C in the Museum of Hunan provincial key laboratory for genetic improvement of domestic animal, Changsha, China. We extracted the total genomic DNA of Niya chicken and used the genomic DNA to generate Illumina sequencing libraries. The libraries then sequenced on Illumina Hiseq 2500 sequencing platform. In total, we generated 11 Gb raw sequencing data and submitted the sequence reads to the NCBI Sequence Read Archive (SRA) with accession number SRR4302067. The assembled complete mitochondrial genome of Niya chicken has been deposited in Genbank with accession number MT635915.

The complete mitogenome sequence of Niya chicken was annotated using MITOS (Bernt et al. 2013) and tRNAscan-SE 2.0 (Chan and Lowe 2019). The mitogenome of Niya is a typical circular DNA molecule of 16,784 bp, including 1 noncoding control region (D-loop region), 22 transfer RNA genes (tRNAs), 2 ribosomal RNA genes (rRNAs), and 13 protein-coding genes (PCGs). The 22 tRNA genes are scattered among PCGs and rRNAs. Most genes including 2 rRNAs, 12 PCGs and 14 tRNAs are encoded on the heavy strand. Only one PCG (*ND6*) and the rest 8 tRNAs are encoded on the light strand. Twelve of 13 PCGs initiate with an ATG start codon except for *COX1*, which begins with GTG. The PCGs use four types of stop codons which are TAA, TAG, AGG and incomplete stop codon T, which dues to the 5′ terminal of the adjacent gene (Anderson et al. 1981).

To investigate the maternal phylogenetic position of Niya chicken, we retrieved the complete mitochondrial genome sequences of different chicken breeds from Genbank and built a neighbor-joining (NJ) phylogenetic tree by using Mega 7.0 (Kumar et al. 2016) with 1000 bootstrap replicates. From the NJ tree (Figure 1), we found the Niya chicken is close related with Tulufan, Tibetan and Lverwu. However, Zhuxiang has the furthest genetic distance with Niya chicken. Our work provides a valuable source of data for phylogenetic studies and helps us to understand the maternal origins of the native chickens.

**Figure 1.**
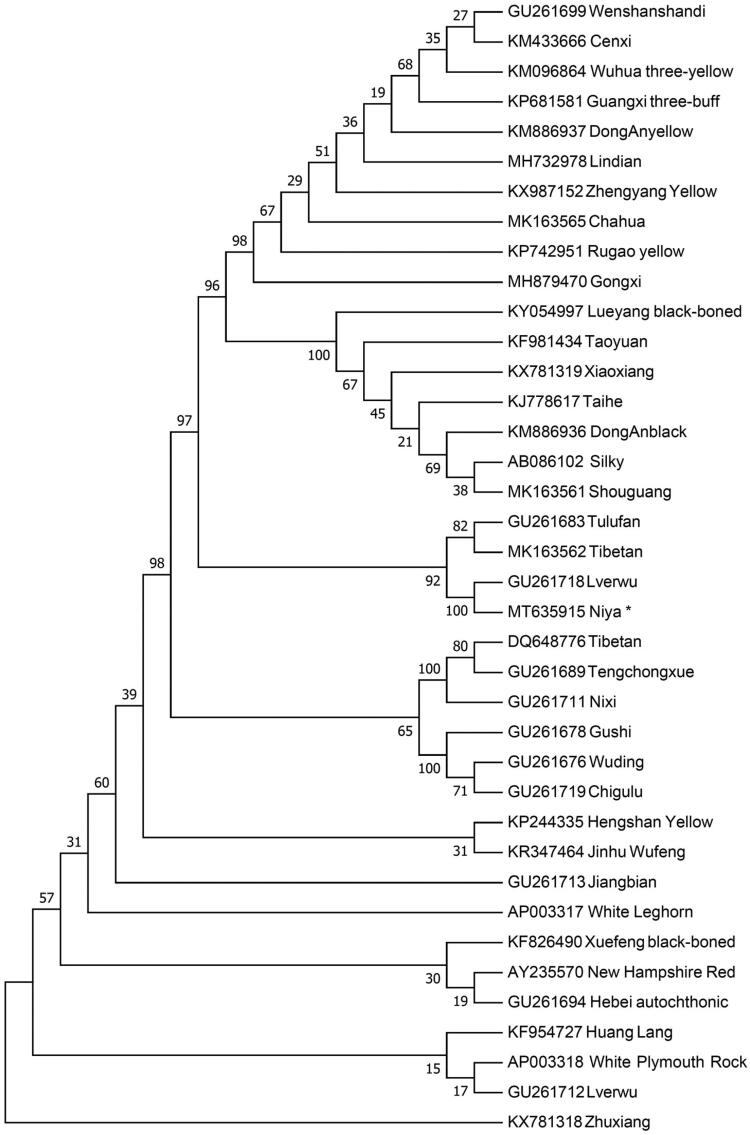
Neighbor-joining tree based on the complete mitochondrial DNA sequence of 38 chicken breeds. GenBank accession numbers are given before the species name.

## Data Availability

The sequence data that support the findings of this study are openly available in the NCBI Sequence Read Archive (SRA) at http://www.ncbi.nlm.nih.gov/sra/ with accession number SRR4302067. The complete mitochondrial genome of Niya chicken (Gallus gallus) is openly available in GenBank at http://www.ncbi.nlm.nih.gov/genbank with accession number MT635915.
